# Artificial intelligence in endometrial cancer: multimodal deep learning and future perspectives in precision gynecologic oncology — a structured narrative review

**DOI:** 10.3389/fonc.2026.1910139

**Published:** 2026-09-07

**Authors:** Erik Lajtman, Michaela Miškovičová, Peter Dobročan, Alexandra Pôbišová, Rastislav Sysák

**Affiliations:** 1 Department of Gynecology and Obstetrics, Faculty Hospital Nitra, Nitra, Slovakia; 2 Faculty of Social Sciences and Health Care, Constantine the Philosopher University in Nitra, Nitra, Slovakia; 3 Department of Health Studies, The College of Polytechnics Jihlava, Jihlava, Czechia; 4 Department of Oncology, Faculty Hospital Nitra, Nitra, Slovakia; 5 Department of Oncology, First Faculty of Medicine of Charles University and General University Hospital in Prague, Prague, Czechia; 6 Department of Laboratory Medicine, Faculty Hospital Nitra, Nitra, Slovakia; 7 1st Department of Gynecology and Obstetrics, Faculty of Medicine, Comenius University Bratislava, Bratislava, Slovakia

**Keywords:** artificial intelligence, deep learning, endometrial cancer, multimodal AI, precision oncology, radiomics, whole-slide imaging

## Abstract

Endometrial cancer (EC) is the most common gynecologic malignancy in developed countries, with a rising incidence driven by obesity, metabolic syndrome, diabetes mellitus, and aging populations. Artificial intelligence (AI) has rapidly advanced in gynecologic oncology, particularly in diagnostic imaging, digital pathology, radiomics, and multimodal prognostic modeling. Recent breakthroughs in machine learning, deep learning, and whole-slide imaging (WSI), including multimodal prognostic architectures such as HECTOR and explainable AI (XAI), have enabled the development of sophisticated diagnostic and prognostic systems. These systems can differentiate benign from malignant lesions, predict myometrial invasion and lymph node metastases, stratify recurrence risk, and support individualized therapeutic decision-making. Notably, recent multimodal deep-learning models, particularly the HECTOR framework, have demonstrated improved prognostic performance compared with conventional clinicopathologic risk-stratification systems for predicting distant recurrence in externally validated cohorts, although further prospective validation remains necessary. Despite these promising advances, current evidence remains limited by predominantly retrospective study designs, relatively small and frequently imbalanced datasets, potential overfitting of AI models, heterogeneous imaging protocols, limited external validation, and insufficient prospective clinical evidence. These limitations restrict model generalizability across different populations and healthcare settings. Furthermore, ethical, regulatory, and data-governance challenges remain important barriers to widespread clinical implementation. This structured narrative review summarizes AI applications in endometrial cancer, focusing on ultrasound imaging, radiomics, digital pathology, multimodal deep-learning, explainable AI, prognostic modeling, clinical translation, and future perspectives in personalized care.

## Introduction

Endometrial cancer (EC) is the most prevalent gynecologic malignancy in developed countries and poses a significant global health challenge (1, 2). Its incidence is continuously rising worldwide, primarily driven by the increasing prevalence of obesity, insulin resistance, diabetes mellitus, metabolic syndrome, and extended life expectancy (1, 3). While most patients are diagnosed at an early stage and experience favorable outcomes, advanced-stage and recurrent EC are associated with significantly poorer prognoses and limited therapeutic options.

Reliable preoperative assessment and individualized risk stratification are crucial for treatment planning and prognostic evaluation. Traditional diagnostic methods include transvaginal ultrasound, magnetic resonance imaging (MRI), histopathologic examination, and increasingly, molecular classification systems (4, 5).

Recent advances in deep learning, whole-slide imaging (WSI), and multimodal AI frameworks have accelerated the development of precision oncology. The emergence of explainable AI (XAI) systems and multimodal architectures, which integrate histopathology, imaging, molecular profiling, and clinical data into unified prognostic platforms, has been particularly significant (6–9).

Artificial intelligence has rapidly transformed modern oncologic diagnostics and medical imaging (10–12). AI-based systems can recognize complex imaging patterns, extracting radiomic biomarkers, integrating multimodal datasets, and generating predictive models with diagnostic and prognostic applications. In endometrial cancer, AI applications have expanded from lesion classification to predicting myometrial invasion, lymphovascular space invasion (LVSI), lymph node metastases, identifying molecular subtypes, assessing recurrence-risk, and guiding individualized therapeutic strategies (4, 6, 7).

## Methods and literature search

A structured narrative literature review was conducted using the PubMed, Scopus, and Web of Science databases to identify and synthesize the available evidence on the application of artificial intelligence (AI) in endometrial cancer. The literature search included articles published between January 2017 and May 2026 and was limited to publications in English. The literature search was last updated in May 2026.

The narrative synthesis incorporated 36 references selected for their clinical relevance and contribution to the principal thematic areas of the review.

The search strategy combined controlled vocabulary (when applicable), database-specific indexing terms, and free-text keywords using Boolean operators (AND/OR). The search strategy was adapted to the indexing system of each database. The principal search terms included “artificial intelligence”, “machine learning”, “deep learning”, “radiomics”, “whole-slide imaging”, “digital pathology”, “ultrasound”, “magnetic resonance imaging (MRI)”, “explainable artificial intelligence”, “multimodal AI”, and “endometrial cancer”.

Titles and abstracts were initially screened for relevance, followed by full-text assessment of potentially eligible articles. In addition, the reference lists of key publications were manually reviewed to identify further relevant studies that were not captured during the primary database search.

Original research articles, systematic reviews, meta-analyses, clinically relevant translational studies, and validation studies focusing on AI applications in endometrial cancer were considered for inclusion. Eligible studies included those addressing ultrasound imaging, hysteroscopy, magnetic resonance imaging, radiomics, digital pathology, whole-slide imaging, explainable AI, multimodal deep-learning models, and AI-assisted prognostic prediction in endometrial cancer. Editorials, letters to the editor, conference abstracts lacking sufficient methodological detail, case reports, and non-English publications were excluded.

Study selection and evidence synthesis were based on clinical relevance, methodological quality, originality, and translational applicability to gynecologic oncology. Emphasis was placed on recent studies evaluating clinically applicable AI models, external validation, explainability, multimodal architecture, and precision oncology. Landmark publications describing major technological advances in deep learning, radiomics, computational pathology, and AI-assisted diagnostic systems were also included to provide appropriate scientific context.

Because the available evidence is highly heterogeneous with respect to study design, patient populations, imaging modalities, AI algorithms, outcome measures, and validation strategies, quantitative synthesis or meta-analysis was not considered appropriate. Instead, the retrieved evidence was synthesized using a structured narrative approach and organized into thematic sections covering ultrasound imaging, hysteroscopy, MRI radiomics, digital pathology, whole-slide imaging, multimodal artificial intelligence, explainable AI, surgical applications, clinical translation, current challenges, and future perspectives.

Accordingly, the objective of this review was not to provide pooled quantitative estimates, but to critically summarize current evidence, identify emerging trends, discuss methodological limitations, and outline future research directions in AI-assisted diagnosis, prognostic assessment, and clinical management of endometrial cancer. A structured narrative review was considered the most appropriate methodology because of the rapidly evolving nature of AI research in endometrial cancer and the substantial methodological heterogeneity of the available literature. This methodological approach enhanced the transparency and reproducibility of the literature search and evidence synthesis while enabling a comprehensive evaluation of emerging AI technologies and a critical appraisal of their current clinical applicability, limitations, and future perspectives in precision gynecologic oncology.

## Fundamentals of artificial intelligence in oncology

Artificial intelligence involves computational systems that perform tasks traditionally associated with human cognition, such as pattern recognition, classification, predictive modeling, image interpretation, and data integration. Machine learning (ML) is a frequently used AI approach in oncologic diagnostics (13), employing algorithms such as support vector machines, random forests, logistic regression, adaptive boosting, and decision-tree models.

ML approaches are particularly effective for lesion classification, prognostic prediction, radiomic analysis, and biomarker discovery. Deep-learning (DL) methodologies have significantly expanded AI capabilities in oncology, with architectures such as convolutional neural networks (CNNs) and transformers enabling automated extraction of complex imaging features directly from raw datasets, eliminating the need for predefined handcrafted parameters (10, 11).

**Figure 1:**
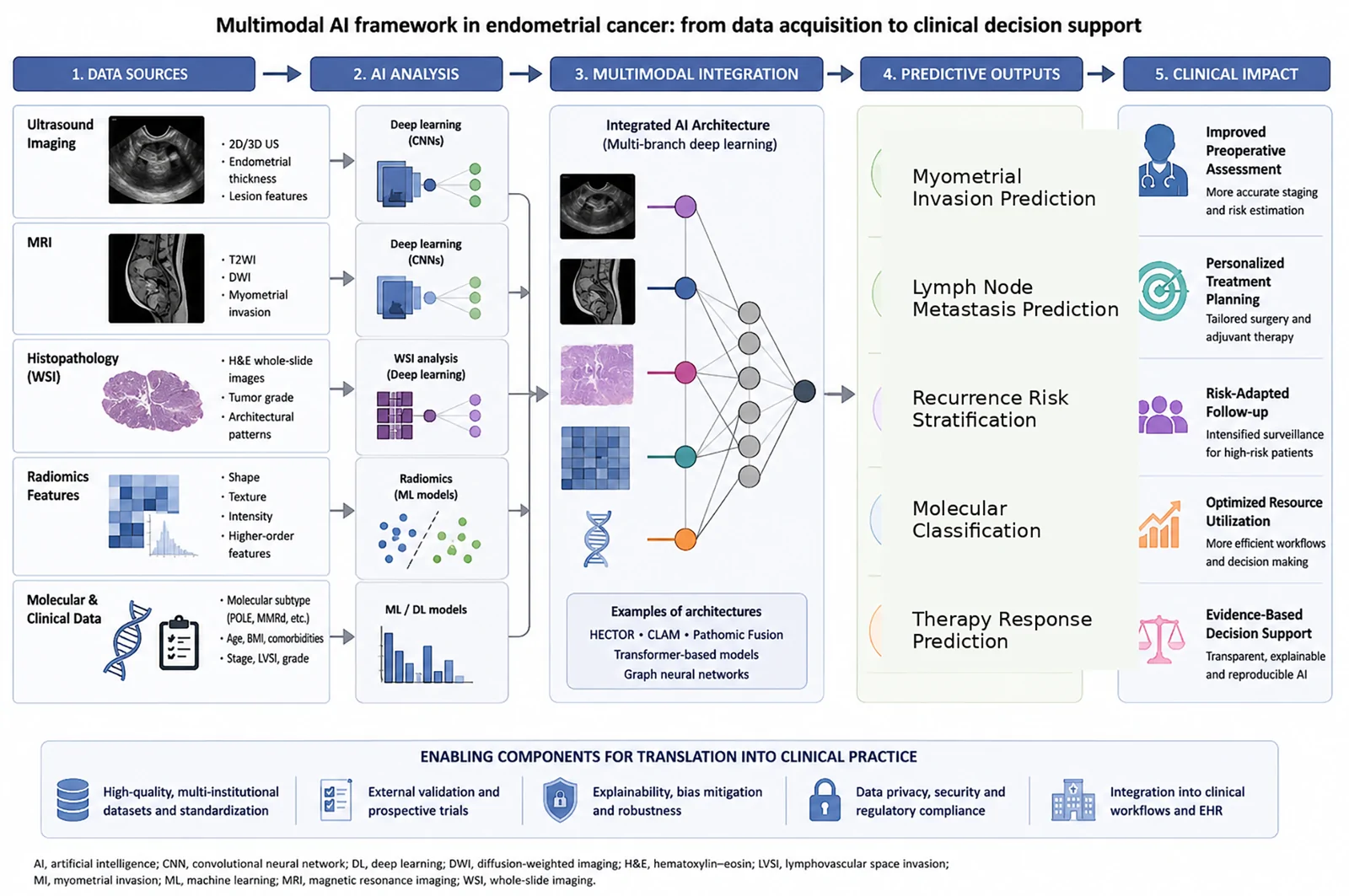
Multimodal artificial intelligence framework in endometrial cancer. Ultrasound imaging, magnetic resonance imaging, whole-slide histopathology, radiomics, molecular profiling, and clinical variables are processed using machine-learning and deep-learning algorithms and integrated into multimodal predictive architectures. These systems support prediction of myometrial invasion, lymph node metastasis, molecular subtype, recurrence risk, and treatment response, facilitating personalized clinical decision-making and precision oncology.

DL systems have shown promising results in ultrasound interpretation, MRI analysis, lesion segmentation, histopathologic evaluation, and digital pathology (10, 14), as illustrated in **Figure 1**. Modern AI increasingly leverages whole-slide imaging (WSI) analysis of digitized hematoxylin-and-eosin (H&E)-stained slides, enabling the extraction of prognostically relevant histomorphology patterns often missed by conventional pathologic assessment.

Radiomics is another rapidly evolving field, extracting quantitative imaging biomarkers that reflect tumor heterogeneity, vascularization, tissue architecture, cellular density, and biologic aggressiveness (11, 15). In endometrial oncology, radiomics has been investigated for predicting deep myometrial invasion, lymph node metastases, histologic subtype, recurrence risk, molecular classification, and treatment response.

Explainable artificial intelligence (XAI) is a crucial recent development aimed at improving the transparency and interpretability of AI-generated predictions. Methods such as SHAP analysis (SHapley Additive exPlanations), integrated gradients, Grad-CAM visualization, and attention heatmaps identify the regions and variables contributing most to algorithmic outputs (13, 16). Enhanced interpretability may increase clinician confidence, facilitate regulatory approval, and promote safer clinical implementation of AI-based systems. Recent studies also underscore the growing role of AI-assisted ultrasound interpretation, digital pathology, and multiomics integration in precision gynecologic oncology (16) (**Table 1**).

**Table 1:** Representative studies investigating artificial intelligence applications in endometrial cancer: comparison of methodologies, validation strategies, strengths, and limitations.

Study	Modality/input data	AI approach	Clinical task	Cohort/dataset	Main performance	Validation	Main strengths/clinical relevance	Main limitations
Wang et al. (2025) (9)	Gynecologic ultrasound	Modified YOLOv8 deep-learning model	Detection of EC on ultrasound	877 patients; 614 training, 175 validation, 88 testing	Test AUC 0.858; accuracy 85.8%; sensitivity 77.3%; specificity 90.1%	Internal test set	Potential diagnostic support for less-experienced ultrasound physicians; outperformed endometrial thickness alone	Single-center dataset; only histologically confirmed EC cases included, limiting applicability to primary screening populations
Zhang et al. (2021) (20)	Hysteroscopic images	Fine-tuned VGGNet-16 CNN	Classification of endometrial lesions	454 patients; 1,851 images; 250-image test set	Benign vs premalignant/malignant: AUC 0.944; accuracy 90.8%; sensitivity 83.0%; specificity 96.0%	Internal test set	Performance slightly exceeded that of gynecologists; model assistance improved diagnostic accuracy	Retrospective single-center study; static images from one endoscopic system; no prospective/external validation
Saida et al. (2025) (7)	MRI/radiomics	ML and DL approaches	Prediction of myometrial invasion, LVSI, molecular characteristics, and other prognostic features	Review of published MRI-based AI studies	AUC approximately 0.80-0.84 for selected preoperative tasks; up to 0.94 for MSI prediction	Variable across included studies	Demonstrates broad potential of MRI-based radiomics and DL for non-invasive tumor characterization and risk assessment	Review rather than a single validation cohort; substantial heterogeneity in imaging protocols, segmentation, models, and validation
Makris et al. (2017) (22)	Endometrial cytology	Multilayer perceptron ANN	Classification of benign vs malignant endometrial nuclei	Cytologic image dataset	Accuracy up to 95.9%	Internal	Early demonstration of automated endometrial cytologic classification	Limited dataset and lack of robust external validation
Li et al. (2022) (23)	Liquid-based endometrial cytology	U-Net segmentation + DenseNet classification	Automated recognition of benign and malignant endometrial cell clusters	113 patients; 15,913 annotated images; 39,000 segmented cell-cluster patches	Accuracy 93.5%; sensitivity 92.0%; specificity 92.2%; AUC 0.951	Internal test set	Automated segmentation and classification; potential to support cytologic screening and reduce diagnostic workload	Relatively small single-center cohort; multicenter validation required
Fell et al. (2023) (24)	H&E endometrial-biopsy WSI	Supervised CNN patch classification + slide-level ML classification	Classification of slides as malignant, other/benign, or insufficient	2,909 slides from multiple tissue-processing laboratory settings	Final test accuracy 89.7%; 96.6% of malignant slides correctly classified	Held-out test set including slides from laboratory processing groups excluded from training	Directly applicable to pathology triage and prioritization of malignant biopsies; substantial slide-level dataset	Limited geographic diversity; performance on insufficient specimens weaker; further validation across scanners and institutions required
Song et al. (2022) (8)	H&E WSI	Deep learning	Histologic subtype and tumor-origin classification	Digital histopathology datasets	AUROC 0.944 for endometrioid vs serous EC; 0.939 for endometrial vs endocervical adenocarcinoma	Internal/dataset-based validation	High discrimination using routinely available H&E slides	Retrospective datasets; prospective clinical utility not established
Feng et al. (2023) (25)	H&E WSI	Attention-based multiple-instance learning with ResNet-18 feature extraction	Prediction of lymph-node metastasis	Internal: 564 patients/2,104 WSI; external: 261 patients/533 WSI from two independent institutions	Internal AUC 0.938, sensitivity 83.0%, specificity 91.1%; external AUC 0.770, sensitivity 81.4%, specificity 52.0%	External validation	Outperformed Mayo criteria internally; interpretable heatmaps; clinically relevant LNM risk stratification	Retrospective; based mainly on postoperative resection specimens; substantial performance decline in external validation
Volinsky-Fremond et al. (2024) (12) - HECTOR	H&E WSI + image-predicted molecular class + anatomical stage	Multimodal DL; vision transformer + attention-based MIL	Prediction of distant recurrence and individualized risk stratification	2,072 patients from 8 cohorts, including PORTEC-1/-2/-3; internal test n=353; external tests n=160 and n=151	C-index 0.789 internal; 0.828 and 0.815 external; 10-year distant recurrence-free probability 97.0%, 77.7%, and 58.1% in low-, intermediate-, and high-risk groups	Two external test cohorts	Strong multimodal prognostic performance; outperformed conventional clinicopathological/molecular models; potential prediction of chemotherapy benefit	Requires prospective clinical validation before routine implementation
Erdemoglu et al. (2023) (32)	Clinical variables + endometrial thickness	Multiple ML algorithms; best-performing MLP model	Prediction of EIN/EC risk	564 patients; 451 training and 113 internal validation	AUC 0.938; accuracy 94%; precision 0.71; recall 0.50; F1 score 0.59	Internal validation	Simple inputs (age, BMI, endometrial thickness); potential for accessible individualized risk assessment	No external validation; relatively small number of EIN/EC events; moderate recall despite high overall accuracy

AI, artificial intelligence; ANN, artificial neural network; AUC, area under the receiver operating characteristic curve; BMI, body mass index; CNN, convolutional neural network; DL, deep learning; EC, endometrial cancer; EIN, endometrial intraepithelial neoplasia; FIGO, International Federation of Gynecology and Obstetrics; LNM, lymph node metastasis; LVSI, lymphovascular space invasion; MIL, multiple-instance learning; ML, machine learning; MLP, multilayer perceptron; MRI, magnetic resonance imaging; MSI, microsatellite instability; WSI, whole-slide imaging.

## Artificial intelligence applications in ultrasound imaging

Transvaginal ultrasound (TVS) is the primary imaging modality for evaluating abnormal uterine bleeding and endometrial pathology due to its accessibility, low cost, and noninvasive nature (4, 5). However, its interpretation is highly dependent on operator expertise and image quality. AI-assisted ultrasound offers a promising solution to enhance diagnostic reproducibility (4). A key application is differentiating between benign and malignant endometrial lesions. Deep-learning algorithms analyzing grayscale texture and Doppler vascularization have demonstrated diagnostic performance comparable to that of expert sonographers in selected retrospective studies, although evidence remains limited and heterogeneous (4). Reported AUC values for differentiating benign from malignant endometrial lesions ranged from 0.64 to 0.96 (4). These systems may be particularly valuable in primary care and institutions with limited access to specialists.

Another clinically important application is predicting myometrial invasion, which impacts FIGO (International Federation of Gynaecology and Obstetrics) staging, lymph node assessment, and surgical planning. AI-based models can improve preoperative evaluation by identifying subtle infiltration patterns not readily recognized through visual assessment alone (4). Published studies have reported AUC values ranging from 0.77 to 0.81 for predicting myometrial invasion (4).

Predicting lymph node metastases has also become a major research focus. Several AI models integrating imaging features and clinical parameters have demonstrated promising predictive performance for nodal involvement. Reported AUC values for lymph node metastasis prediction ranged from 0.73 to 0.88 (4). While current evidence is limited by retrospective validation, these approaches could eventually facilitate individualized surgical staging and reduce unnecessary lymphadenectomy-associated morbidity.

Furthermore, AI-based ultrasound can improve the standardization of endometrial-thickness measurements, Doppler evaluation, and assessment of vascular patterns (4). The most commonly used input features include patient age, endometrial thickness, vascularity, and other ultrasound-derived clinical parameters, while machine-learning algorithms remain the predominant modeling approach.

Overall, current evidence consistently supports the potential of AI-based ultrasound to improve diagnostic reproducibility and preoperative risk assessment. However, considerable variability exists among published studies regarding patient populations, image acquisition protocols, AI architectures, and validation strategies. Moreover, most available studies are single-center investigations with relatively small patient cohorts, substantial variability in model development, limited external validation, and heterogeneous reporting of diagnostic performance, making direct comparison between models difficult and highlighting the need for standardized multicenter prospective studies before widespread clinical implementation. In addition, a substantial implementation gap persists between high-performing retrospective AI models and their routine clinical deployment, emphasizing the need for prospective validation, standardized reporting, and seamless integration into clinical workflows (4, 5, 18).

## Artificial intelligence applications in hysteroscopy

Hysteroscopy enables direct visualization of the uterine cavity but remains dependent on operator expertise and interpretation. AI-assisted hysteroscopy may improve diagnostic reproducibility and clinical decision-making (19). A recent systematic review of 15 studies published between 2021 and 2025 showed that current applications primarily involve intracavitary lesion classification, chronic endometritis, and intrauterine adhesions (19).

A key application is the differentiation of benign from malignant endometrial pathology using convolutional neural network (CNN) models applied to static images or hysteroscopic video (19). Zhang et al. developed a deep-learning model with diagnostic performance comparable to that of experienced gynecologists (20). Across published studies, diagnostic accuracy generally exceeded 85%, with the best-performing models achieving an accuracy of 91.6%, sensitivity of 89.3%, specificity of 94.2%, and AUC values of up to 0.97 when hysteroscopic images were combined with clinical data (19). Real-time video-based systems have also demonstrated encouraging performance, including sensitivity of 92–100% for automated detection of endometrial polyps (19).

AI has additionally been investigated for chronic endometritis and intrauterine adhesions, with promising diagnostic and prognostic performance (19).

Importantly, AI-assisted hysteroscopy may improve lesion targeting during biopsy and potentially reduce sampling errors associated with blind endometrial sampling (20, 21). Real-time AI systems could therefore support the detection and targeted assessment of atypical hyperplasia and early-stage endometrial carcinoma (19).

Overall, AI-assisted hysteroscopy shows potential to improve diagnostic accuracy, reproducibility, and intraoperative decision-making. However, most studies remain retrospective, involve relatively small cohorts, and lack external validation, with substantial heterogeneity in image acquisition, AI architectures, and validation strategies. Standardized prospective multicenter studies are therefore required before routine clinical implementation (19).

## Radiomics and artificial intelligence in MRI

Magnetic resonance imaging (MRI) is the key advanced imaging modality for local staging of endometrial cancer (11, 15). It is routinely used to evaluate myometrial invasion, cervical stromal involvement, lymph node status, and extrauterine disease.

MRI radiomics has shown value in predicting deep myometrial invasion, lymphovascular space invasion, histologic subtype, molecular classification, and recurrence risk (10, 15). By extracting quantitative features that capture tumor heterogeneity and biological aggressiveness, AI-assisted MRI can provide information beyond conventional visual assessment. These imaging biomarkers may support non-invasive tumor characterization and individualized risk stratification.

Several deep-learning models have demonstrated promising diagnostic performance for MRI interpretation; however, reported accuracy varies considerably depending on dataset characteristics, imaging protocols, and validation methodology (7, 15). Recent MRI radiomics studies have reported AUC values ranging from approximately 0.80 to 0.84 for predicting deep myometrial invasion, lymphovascular space invasion, and overall preoperative risk stratification, whereas prediction of microsatellite instability (MSI) achieved AUC values of up to 0.94, highlighting the potential of AI-assisted MRI for non-invasive risk assessment and molecular characterization before surgery (7).

Furthermore, several studies have explored multimodal approaches that integrate MRI radiomics with clinical, histopathologic, and molecular data (20). These integrated systems have demonstrated improved predictive performance in selected studies; for example, a multicenter fusion model predicting recurrence risk achieved an AUC of 0.85, outperforming models based on clinicopathologic variables (AUC 0.75) or radiomics alone (AUC 0.78) (7).

Compared with ultrasound-based AI models, MRI-based radiomics offers the advantage of incorporating quantitative tissue characteristics that may improve prediction of tumor aggressiveness and molecular subtype. However, radiomic performance is strongly influenced by scanner variability, image acquisition protocols, tumor segmentation, and feature extraction methods. Nevertheless, prospective multicenter validation and standardized radiomics methodologies remain essential before routine clinical implementation (7).

## Digital pathology, whole-slide imaging, and deep learning

Digital pathology and WSI-based deep-learning systems are among the fastest-growing areas of AI research in endometrial cancer (6–9).

The increasing digitalization of pathology has significantly accelerated the development of AI-assisted histopathologic diagnostics. Conventional microscopy is now complemented by digitally scanned slides and WSI platforms, enabling computational pathology analysis and deep-learning-assisted interpretation (12, 22). This transition has opened new avenues for developing AI systems that can assist in cytologic classification, lesion stratification, and prognostic evaluation (6–8).

Artificial neural-network (ANN) systems have demonstrated good diagnostic performance in distinguishing benign from malignant endometrial nuclei and cytologic lesions (22). Makris et al. demonstrated the feasibility of multilayer perceptron artificial neural networks for computer-assisted endometrial cytology, reporting overall case-classification accuracy of up to 95.9%, thereby providing one of the earliest demonstrations of AI-assisted discrimination between benign and malignant endometrial lesions (22). More recently, Li et al. developed a deep-learning cytopathology system combining U-Net segmentation with DenseNet classification for automated recognition of benign and malignant endometrial cell clusters. In a dataset derived from 113 patients, the model achieved an accuracy of 93.5%, sensitivity of 92.0%, specificity of 92.2%, and an AUC of 0.951, supporting the potential of AI-assisted cytology for endometrial cancer screening and diagnostic workflow optimization (23). Similarly, Fell et al. developed a whole-slide imaging model that correctly classified approximately 90% of endometrial biopsy slides, including 97% of malignant cases, demonstrating its potential to prioritize malignant biopsies in routine pathology workflows (24). In addition, deep-learning models applied to whole-slide histopathology images achieved excellent discrimination of endometrial cancer subtypes, with an AUROC of 0.944 for differentiating endometrioid from serous endometrial carcinoma and an AUROC of 0.939 for distinguishing endometrial from endocervical adenocarcinoma, highlighting the expanding role of AI-assisted digital pathology in histopathologic diagnosis (8). Feng et al. further demonstrated the clinical utility of WSI-based AI by developing a multiple instance-learning model for predicting lymph node metastasis from H&E-stained whole-slide images. Their model achieved an AUC of 0.938, sensitivity of 0.830, and specificity of 0.911 in the internal validation cohort, substantially outperforming the conventional Mayo criteria (AUC 0.666), while maintaining an AUC of 0.770 in external validation (25). These findings highlight the potential of AI-assisted digital pathology to improve preoperative risk stratification and support surgical decision-making regarding lymph node assessment (25).

A major recent advance is the HECTOR (Histopathology-based Endometrial Cancer Tailored Outcome Risk) model introduced by Volinsky-Fremond et al. (12). HECTOR is a multimodal prognostic deep-learning system that integrates H&E whole-slide images, image-based molecular classification, and FIGO anatomic staging. The model demonstrated excellent prognostic performance, achieving C-indices of 0.789, 0.828, and 0.815 in internal and external validation cohorts, outperforming conventional clinicopathologic and molecular risk-stratification approaches. Furthermore, HECTOR stratified patients into low-, intermediate-, and high-risk groups with 10-year distant recurrence-free probabilities of 97.0%, 77.7%, and 58.1%, respectively (12).

Crucially, HECTOR also demonstrated the ability to predict benefit from adjuvant chemotherapy in patients with high-risk endometrial cancer, showing superior predictive performance compared with currently used clinicopathologic and molecular risk-stratification methods in the PORTEC-3 trial (8, 12). These findings suggest that multimodal AI systems could eventually contribute not only to prognostic evaluation but also to individualized therapeutic decision-making (12).

Explainability analysis is another vital aspect of WSI-based deep-learning systems. Integrated-gradient methods and attention heatmaps have revealed that favorable prognostic morphologic features include inflammatory infiltrates, smooth luminal borders, and intraepithelial lymphocytes. Conversely, unfavorable features included LVSI, solid tumor growth, nuclear atypia, desmoplastic stromal reaction, and high mitotic activity (8). Quantitative image analysis further confirmed that low-risk regions were characterized by significantly higher inflammatory-cell density, whereas high-risk regions showed increased mitotic activity and larger tumor nuclei (12).

These observations indicate that modern AI systems can identify biologically relevant morphologic correlates beyond conventional histopathologic classification, thereby enhancing our understanding of tumor biology.

Among currently available AI applications, whole-slide imaging combined with multimodal deep-learning appears to have the strongest evidence for prognostic stratification because several models have undergone external validation. Nevertheless, most systems remain research tools requiring prospective multicenter clinical validation before routine implementation. Prospective evaluation of HECTOR is currently being pursued within the PORTEC-4a trial and other validation cohorts to establish its role in routine clinical practice (12).

## Multimodal artificial intelligence: data fusion, clinical applications, and future perspectives

Compared with unimodal AI systems that rely on a single source of information (e.g., ultrasound, MRI, or histopathology), multimodal deep-learning models integrate complementary data from multiple modalities, including imaging, whole-slide pathology, molecular profiling, laboratory biomarkers, and clinical variables. These models provide a more comprehensive representation of tumor biology and have demonstrated improved prognostic discrimination in selected studies, particularly for recurrence risk prediction. Recent advances indicate that multimodal AI may improve diagnostic accuracy, prognostic stratification, and treatment planning across the continuum of gynecologic cancer care (6). However, direct comparisons between unimodal and multimodal models remain limited because of substantial heterogeneity in study design, datasets, and performance metrics.

Integrating imaging-based molecular prediction into multimodal frameworks is particularly promising. Recent AI systems can predict molecular EC subtypes directly from H&E slides, including POLE-mutated, mismatch-repair-deficient, p53-abnormal, and NSMP tumors (8). These approaches offer the potential to reduce costs while improving the accessibility of molecular classification, particularly in resource-limited settings. The integration of genomic, transcriptomic, proteomic, metabolomic, and epigenomic data may further support biomarker discovery and individualized treatment strategies (17).

Multimodal AI systems employ different data-fusion strategies (26, 27). Early-fusion approaches combine heterogeneous data before model training, whereas late-fusion methods integrate predictions generated independently by individual models. Hybrid fusion strategies combine elements of both approaches, seeking to balance comprehensive information integration with model flexibility and interpretability. Although comparative evidence remains limited, hybrid and late-fusion architectures may offer practical advantages by facilitating the integration of heterogeneous clinical datasets while maintaining greater modularity and interpretability.

Federated learning and privacy-preserving AI systems may further enable collaborative model development across multiple institutions while maintaining patient confidentiality by allowing algorithms to be trained locally without sharing patient-level data. This approach could help address the reliance on retrospective single-center datasets, potentially improving model generalizability, reducing dataset bias, and supporting robust external validation (6).

Future multimodal AI systems should emphasize clinician-centered explainability, standardized evaluation frameworks, and seamless integration into clinical workflows to ensure that AI predictions are not only accurate but also clinically interpretable and actionable (26, 27).

## Explainable artificial intelligence and biological insights

One of the principal challenges limiting the clinical adoption of AI is its “black-box” nature. Consequently, explainable artificial intelligence (XAI) has become a key prerequisite for successful clinical implementation.

Modern explainability methods, including SHAP analysis, integrated gradients, saliency maps, and attention visualization, improve transparency by identifying the imaging features and biological variables that contribute most to AI predictions, thereby increasing clinician confidence in AI-assisted decision-making (13, 16, 26, 27). Recent explainability analyses have demonstrated that AI systems can identify clinically and biologically relevant histomorphologic patterns associated with prognosis, including inflammatory infiltrates, immune activation, lymphovascular space invasion (LVSI), and aggressive tumor architecture (8). These findings strengthen the biological interpretability of AI predictions and support the potential role of AI in biomarker discovery and precision oncology.

Although current evidence suggests that multimodal AI may improve prognostic performance by integrating complementary clinical, imaging, molecular, and pathologic information, increasing model complexity also requires larger annotated datasets, standardized data acquisition, rigorous external validation, and transparent model development. Future prospective multicenter studies directly comparing unimodal and multimodal AI systems will be essential to determine their incremental clinical value and facilitate routine clinical implementation in gynecologic oncology (6, 26, 27).

## Artificial intelligence in surgical navigation and robotic oncology

Artificial intelligence is increasingly being explored as a supportive technology in surgical planning and minimally invasive management of endometrial cancer. Robotic-assisted surgery provides enhanced three-dimensional visualization, instrument articulation, and surgical precision, particularly in patients with obesity or complex pelvic anatomy. The emerging role of AI lies primarily in augmenting surgical navigation, anatomical recognition, and intraoperative decision-making rather than enabling autonomous surgery (28).

AI-assisted surgical navigation may facilitate real-time identification of critical anatomical structures, including the ureters, vessels, nerves, and tissue planes. Computer-vision and deep-learning algorithms applied to intraoperative imaging could improve surgical orientation and potentially reduce the risk of inadvertent injury during complex pelvic procedures. Although much of the current evidence originates from other surgical specialties, these technologies may be transferable to gynecologic oncology, particularly during hysterectomy and lymph-node assessment (28). Emerging robotic platforms are also integrating multimodal imaging, including CT, MRI, intraoperative ultrasound, and AI-based tissue recognition, providing additional opportunities for real-time anatomical guidance (29).

AI may also contribute to intraoperative tissue and lymph-node characterization. In a recent prospective study of 172 sentinel lymph nodes from 63 patients with gynecologic malignancies, ex vivo shear-wave elastography was rapid and reproducible but demonstrated limited diagnostic performance when used alone (AUC 0.53, sensitivity 44%, and specificity 74%) (30). These findings suggest that isolated imaging parameters may be insufficient for reliable intraoperative classification and support further investigation of approaches integrating imaging features with radiomics, machine-learning algorithms, and clinicopathological data (30).

The integration of AI with robotic surgery could support intelligent surgical platforms that combine real-time image analysis, anatomical recognition, automated feedback, and patient-specific data under surgeon supervision. However, AI-assisted robotic oncology is still in the early stages of clinical development. Current evidence is mainly based on feasibility studies, small cohorts, and findings extrapolated from other surgical fields. Therefore, prospective multicenter studies showing meaningful improvements in surgical outcomes are needed before these technologies can be widely adopted in endometrial cancer surgery (28).

## Clinical translation, validation, and current challenges

Despite remarkable technological advances, routine clinical implementation of AI in endometrial cancer remains limited. Most available models have been developed using relatively small, retrospective single-center datasets, with insufficient external validation and substantial methodological heterogeneity (4, 18, 31–33). Performance may decline during external validation because of dataset bias, protocol heterogeneity, institutional variability, and domain shift, highlighting the need for standardized methodologies and prospective multicenter validation across diverse populations.

Dataset imbalance further limits generalizability. Available datasets are frequently dominated by early-stage endometrioid cancers, whereas high-grade histologic subtypes, non-endometrioid tumors, and advanced-stage disease remain underrepresented. Future studies should therefore prioritize large, balanced multicenter datasets representing diverse histologic subtypes, disease stages, populations, and healthcare environments (6, 31). In radiomics, additional variability related to image acquisition, scanner platforms, segmentation, and feature extraction further challenges reproducibility.

Importantly, high diagnostic or prognostic performance does not necessarily translate into improved patient outcomes. Most studies evaluate surrogate endpoints rather than clinically meaningful outcomes such as survival, treatment selection, quality of life, or cost-effectiveness. Prospective studies demonstrating tangible clinical benefit are therefore essential before widespread implementation.

Successful clinical translation also requires integration with hospital information systems, radiology and pathology platforms, electronic health records, and routine clinical workflows (34). AI should function as a clinical decision-support tool that complements rather than replaces physician expertise (16). Transparent and interpretable outputs, user-friendly interfaces, and appropriate clinician training are essential, particularly because many deep-learning models remain only partially explainable despite advances in XAI (13, 16).

Ethical, regulatory, and economic challenges include data privacy, algorithmic bias, cybersecurity, legal responsibility, patient consent, data ownership, reimbursement, and regulatory oversight (35, 36). Evidence regarding cost-effectiveness also remains limited, while implementation requires substantial investment in digital infrastructure, data management, personnel training, and long-term system maintenance (6).

These barriers may be particularly relevant for multimodal AI, which requires access to multiple high-quality data sources, including advanced imaging, digital pathology, molecular testing, electronic health records, and adequate computational infrastructure. Limited availability of these resources may restrict implementation, particularly in resource-limited healthcare settings. Scalable approaches capable of maintaining robust performance with heterogeneous or incomplete clinical data will therefore be important for equitable adoption.

Overall, successful translation of AI into routine endometrial cancer care will require prospective multicenter validation, standardized methodologies, representative datasets, clinically meaningful outcome assessment, transparent and interpretable models, appropriate regulatory oversight, demonstrated cost-effectiveness, and seamless integration into clinical workflows.

## Conclusion

Artificial intelligence is rapidly reshaping the diagnosis and management of endometrial cancer, with promising applications across imaging, digital pathology, hysteroscopy, molecular profiling, and multimodal data integration. AI-assisted systems have demonstrated encouraging performance in lesion classification, prediction of myometrial invasion, recurrence risk assessment, and individualized risk stratification. Recent advances in whole-slide image analysis and multimodal architecture further highlight the potential of AI to support precision gynecologic oncology.

Despite these advances, translation into routine clinical practice remains limited by insufficient prospective multicenter validation, methodological heterogeneity, limited generalizability, and unresolved challenges related to interpretability, workflow integration, regulation, and cost-effectiveness.

AI should therefore be regarded as a clinical decision-support tool that complements rather than replaces expert judgment. Its integration with clinical expertise may improve diagnostic consistency, refine risk stratification and treatment planning, and support more personalized patient care while maintaining physician oversight of complex clinical decisions.

Future progress will depend on robust multicenter validation, standardized evaluation frameworks, and the development of transparent and clinically integrated AI systems. Addressing these priorities will be essential to translate current technological advances into meaningful improvements in the management of patients with endometrial cancer.
